# Oxidized primary arc magmas: Constraints from Cu/Zr systematics in global arc volcanics

**DOI:** 10.1126/sciadv.abk0718

**Published:** 2022-03-23

**Authors:** Si-Yu Zhao, Alexandra Yang Yang, Charles H. Langmuir, Tai-Ping Zhao

**Affiliations:** 1 State Key Laboratory of Isotope Geochemistry, Guangzhou Institute of Geochemistry, Chinese Academy of Sciences, Guangzhou 510640, China.; 2 CAS Center for Excellence in Deep Earth Science, Guangzhou, China.; 3 Southern Marine Science and Engineering Guangdong Laboratory (Guangzhou), Guangzhou, China.; 4 University of the Chinese Academy of Sciences, Beijing 100049, China.; 5 Harvard University, Cambridge, MA, USA.; 6 Key Laboratory of Mineralogy and Metallogeny, Guangzhou Institute of Geochemistry, Chinese Academy of Sciences, Guangzhou 510640, China.

## Abstract

Arc volcanics are more oxidized than mid-ocean ridge basalts (MORB), but it is debated whether this is a mantle feature or a result of magmatic evolution. Copper, a sulfur-loving element, has been used to trace the behavior of redox-sensitive sulfur during mantle melting and infer similar redox states of sub-arc and sub-ridge mantle. Previous studies, however, neglected elevated sulfur contents in the sub-arc mantle, leading to underestimation of oxygen fugacities, and did not recognize systematic Cu variations in arc volcanics. Here, we show that the Cu/Zr ratio is a sensitive indicator that responds to sulfur content, oxygen fugacity, and extent of melting of the mantle. Because of higher mantle S contents, Cu systematics of arc magmas require one log unit higher oxygen fugacities of sub-arc than sub-ridge mantle. Low Cu contents of thick-crusted arc volcanics result from low extents of melting of sulfur-rich mantle, obviating the need for deep crustal sulfide fractionation, with substantial implications for the origin of porphyry-Cu deposits.

## Teaser

Similar Cu systematics for primary arc and MORB magma result from more oxidized sub-arc mantle with higher sulfur from slab flux.

## INTRODUCTION

Oxygen fugacity (*f*o_2_) in magmatic system is used to decipher the evolution of the Earth’s interior and exterior, mantle redox states, mass exchanges during subduction, and the formation of Cu-porphyry ore deposits (*1*–*11*). Arc volcanics are more oxidized than mid-ocean ridge basalts (MORB) (*2*, *12*), but it has been widely debated whether higher *f*o_2_ results from mantle (*2*, *8*, *13*–*17*) or crustal processes (*10*, *11*, *18*, *19*). Measurements of Fe^3+^/ΣFe of arc volcanics by micro–x-ray absorption near-edge structure (μ-XANES) spectroscopy show that arc basalts are more oxidized than MORB, suggested to be inherited from their mantle sources (*2*, *8*, *12*–*15*). Arc magmas inevitably experience differentiation at crustal levels, however, leading to suggestions that more oxidized arc magmas result from crustal differentiation process (*10*, *18*, *19*).

To resolve the debate, multivalent elements sensitive to redox conditions have been used to track the redox states of the mantle (*10*, *18*, *19*). Sulfur, in particular, is sensitive to variations in oxygen fugacities. At *f*o_2_ of FMQ (the fayalite-magnetite-quartz buffer), typical of average MORB magmas (*20*–*22*), the solubility of S (mostly in the form of S^2−^) in basaltic magma is ~1200 parts per million (ppm) (*23*–*25*). At higher *f*o_2_ (>FMQ + 1.5), the solubility of S (mostly in the form of S^6+^) in basaltic magma increases substantially to ~1 weight % (wt %) (*23*–*25*). Such a remarkable increase of sulfur solubility in magma at higher *f*o_2_ would accelerate the efficiency of sulfur extraction during mantle melting and lead to higher contents of sulfur and sulfur-loving elements (e.g., Cu) in the melts for a given extent of melting (*10*), which enables the Cu contents of primary arc magma to be used as an index for mantle redox states (*10*). On this basis, the similar range of Cu contents in relatively primitive arc volcanics and ocean ridge basalts has been used to argue for similar mantle oxygen fugacities during melting (*10*).

A problem with this conclusion is that Cu concentrations in basaltic magma are controlled not only by oxygen fugacity but also by the sulfur contents in the mantle source, neglected in previous treatments (*10*). The partition of Cu during mantle melting is dominantly controlled by the behavior of sulfide in the mantle as Cu is highly compatible in sulfides ($D_{\text{Cu}}^{\text{sulfide}/\text{melt}}$ ranges from 200 to 1600) (*26*, *27*) and incompatible in silicate minerals ($D_{\text{Cu}}^{\text{ol}/\text{melt}}$ = 0.05, $D_{\text{Cu}}^{\text{opx}/\text{melt}}$ = 0.06, $D_{\text{Cu}}^{\text{cpx}/\text{melt}}$ = 0.039, $D_{\text{Cu}}^{\text{spinel}/\text{melt}}$ = 0.19, and $D_{\text{Cu}}^{\text{garnet}/\text{melt}}$ = 0.042) (*28*). Thus, as long as there is a residual sulfide phase (see Materials and Methods), Cu concentrations in the melt are controlled by a bulk partition coefficient of Cu (bulk *D*_Cu_) and the proportion of residual sulfide (*X*_sulfide_)

$$\begin{array}{c} \text{bulk}\;D_{\text{Cu}}=X_{\text{olivine}}*D_{\text{Cu}}^{\text{ol}/\text{melt}}+X_{\text{orthopyroxene}}*D_{\text{Cu}}^{\text{opx}/\text{melt}}+X_{\text{clinopyroxene}}*\\ D_{\text{Cu}}^{\text{cpx}/\text{melt}}+X_{\text{spinel}(\text{garnet})}*D_{\text{Cu}}^{\text{spinel}(\text{garnet})/\text{melt}}+X_{\text{sulfide}}*D_{\text{Cu}}^{\text{sulfide}/\text{melt}} \end{array}$$ (1)

$$X_{\text{sulfide}}=C_{S}^{\text{mantle}}/C_{S}^{\text{sulfide}}$$ (2)

where *X* is the mass portion of a particular mineral in the mantle assemblage and the sulfur content in mantle sulfides ($C_{S}^{\text{sulfide}}$) of 36.9 wt % is used according to Jugo *et al*. (*24*). Higher sulfur contents ($C_{S}^{\text{mantle}}$) increase the portion of sulfide phases (*X*_sulfide_) in the mantle, which increases the bulk partition coefficient of Cu during melting (bulk *D*_Cu_; Eqs. 1 and 2), resulting in lower Cu contents in primary arc magma at a given melting degree and *f*o_2_. Higher S and higher *f*o_2_ thus have offsetting effects on Cu contents in mantle melts, and both must be considered.

**Fig. 1.**
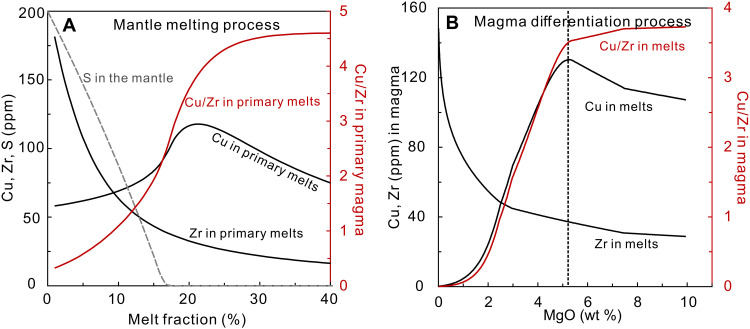
Cu, Zr and S systematics during magmatic processes. Cu, Zr, and Cu/Zr variations during (**A**) partial melting of the depleted mantle and (**B**) magma differentiation of a primitive basalt from the Mariana arc (HPD1147R16) (*82*). The black dashed line in (B) indicates the sulfide-saturated fractionation toward lower MgO contents. (A) During mantle melting, Cu contents increase with higher extents of partial melting (*F*) and then decrease shortly after sulfide exhaustion; however, Cu/Zr ratios would increase with *F* and stay constant shortly after sulfide exhaustion (table S2). Therefore, the behavior of sulfur during mantle melting is well recorded by Cu/Zr ratios relative to Zr contents in primary magmas. The modeling parameters can be found in Materials and Methods for melting beneath mid-ocean ridges. (B) Cu contents in melts increase during initial sulfide-undersaturated differentiation, but Cu/Zr ratios remain relatively constant before sulfide saturation (>6 wt % MgO) and thus would record primary arc magma signatures (table S3).

A second problem is that the absolute Cu contents of mantle melts, including arc volcanics, vary with the extents of partial melting and fractional crystallization (Fig. 1). During mantle melting, the mass portion of sulfide phase in the mantle would gradually decrease with progressive melting, resulting in decreasing bulk partition coefficient of Cu (Eq. 1) and increasing Cu contents in the melt. When sulfides are fully extracted from the mantle, the bulk *D*_Cu_ reaches the minimum values (~0.05), which highly accelerates Cu extraction to the melt, and leads to the maximum Cu contents in the melt shortly after sulfide exhaustion (Fig. 1A and fig. S1). Further melting would then result in dilution of Cu in the melt. While this behavior was recognized by Lee *et al*. (*10*), they did not consider that differences in Cu contents in relatively primitive volcanics from one tectonic setting to another could depend on differences in extents of melting rather than redox states.

A third problem is the estimation of Cu contents in primary arc magmas. Sulfide-undersaturated fractionation of basaltic magma would increase Cu contents in the melt (Fig. 1), and to avoid such an effect, Lee *et al*. (*10*) used arc volcanics and MORB with MgO >8 wt % to represent primary magma compositions. Comparable Cu ranges of the two groups of rocks thus lead Lee *et al*. (*10*) to argue that arc and MORB magma are comparable in the redox states of their mantle sources. Such a conclusion, however, is based on a small and not necessarily representative sample selection. Only 3% of global arc volcanics have MgO >8 wt % compared to ~37% of MORB at the same MgO range (*29*), and 12 of 37 arcs do not have any volcanic samples with MgO over 8 wt % (table S1; arc data compilation can be found in Materials and Methods). Mg# [mole fractions of 100 * Mg/(Mg + Fe)] is also an alternative index of fractionation, and many arc volcanics with MgO <8 wt % nonetheless have high Mg# over 60 (fig. S3). Therefore, the high MgO arc volcanics are not necessarily parental to the most abundant arc lavas, and we first turn to an evaluation of how to have a more inclusive understanding of Cu variations in arc magmas and then to explore their implications for redox states in the sub-arc mantle.

## RESULTS AND DISCUSSION

### Cu systematics in ocean ridge and arc basalts

**Fig. 2.**
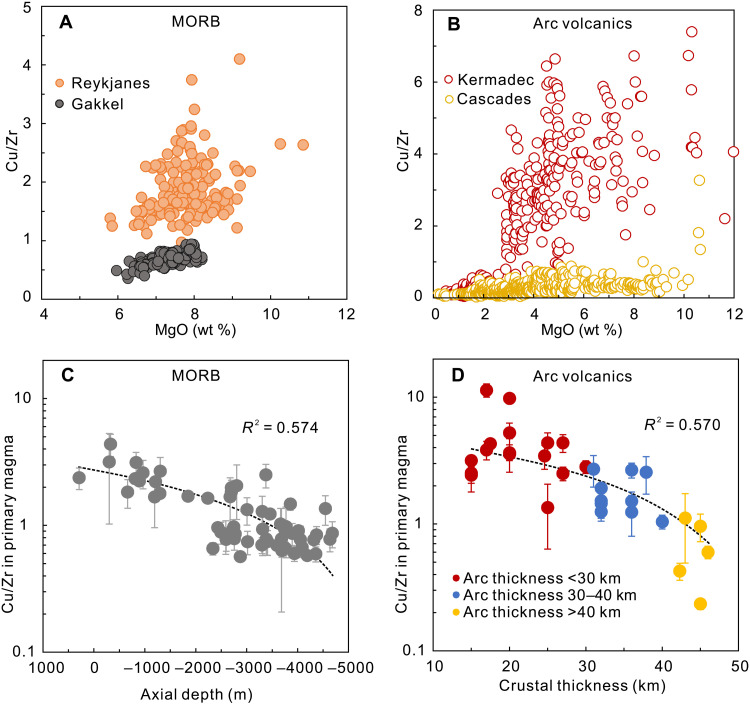
Control of extents of mantle melting on Cu/Zr ratios for MORB and arc volcanics. Cu/Zr systematics for MORB (**A** and **C**) and arc volcanics (**B** and **D**). (A and B) Cu/Zr versus MgO variations for (A) MORB from the Reykjanes Ridge and −6°E to 2°E Gakkel Ridge and for (B) arc volcanics from Kermadec (thin-crusted) and Cascades (thick-crusted) arcs, representing the high and low melting degree endmembers for global MORB and arc volcanics, respectively (fig. S4). It is clearly shown that both MORB and arc volcanics formed by higher degree of partial melting have higher Cu/Zr ratios. (C and D) Cu/Zr ratios in primary MORB and arc magma for individual ridge and arc segments correlate negatively with axial depth for ridges and crustal thickness for arcs, respectively. Gray circles in (C) represent the mean values of Cu/Zr of MORB with MgO over 8 wt % for MORB segments with 2 SE. Colored circles in (D) represent the mean values of Cu/Zr ratios in arc volcanics with MgO over 6 wt % with 2 SE for arc segments with different crustal thickness. MORB data are from Gale *et al*. (*29*) and Yang *et al*. (*83*). The subdivision of ridge and arc segments mainly follows Gale *et al*. (*33*) and Turner and Langmuir (*36*). The axial depth of ridges and crustal thickness are from Gale *et al*. (*29*) and Turner and Langmuir (*36*), respectively.

**Fig. 3.**
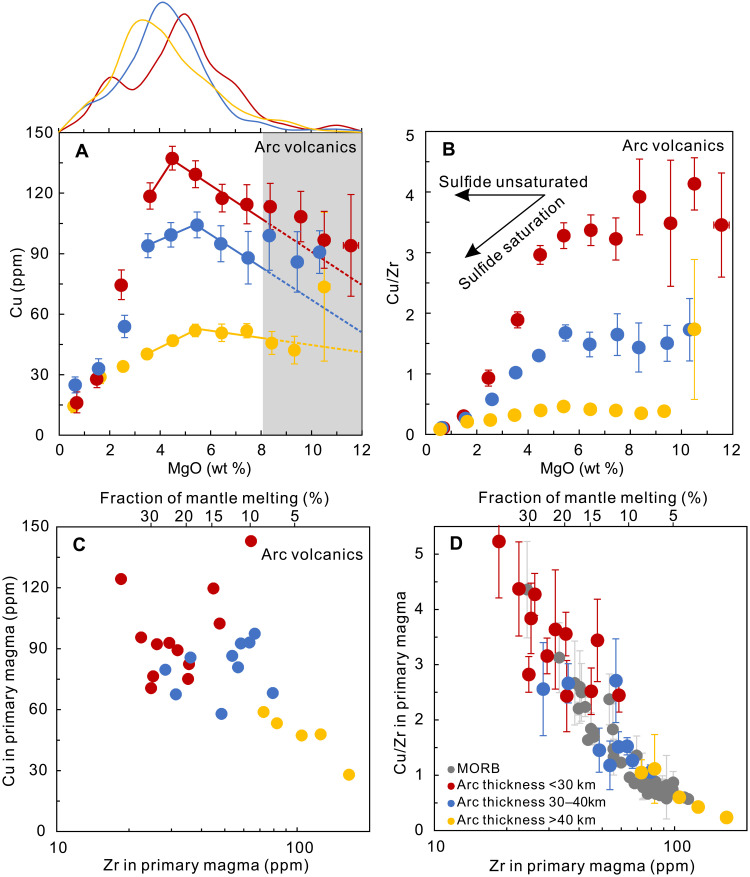
Cu and Cu/Zr systematics in primary arc magma. Copper contents and Cu/Zr ratios versus MgO in arc volcanics (**A** and **B**) and the variation of Cu contents and Cu/Zr ratios versus Zr contents in primary arc magma (**C** and **D**). Individual dots in (A) and (B) represent the mean values of Cu, Cu/Zr, and MgO contents of 1 wt % MgO intervals with 2 SE for global arc volcanics with varied crustal thickness as shown in Fig. 2. (A) Top of the panel represents the probability distribution of MgO contents for arc volcanics with varied crustal thickness. Colored lines suggest that arc volcanics from thinner arcs have systematically higher Cu contents than those from thicker arcs for samples with MgO of 3 to 8 wt %, which represent the majority of arc samples. The dashed extensions of the colored lines represent the inferred Cu contents for primary arc magma with different crustal thicknesses. (B) Cu/Zr ratios in arc volcanics with MgO over 6 wt % are constant before sulfide saturation, which therefore could record the ratios in the primary arc magma. (C) Volcanics from thick arcs are formed by lower degrees of partial melting, with lower Cu contents in primary arc magmas (Cu_90_, corrected Cu contents for magma in equilibrium with mantle olivine of Fo_90_; see Materials and Methods) than those from thick arcs. (D) Cu/Zr ratios in primary arc and MORB magma vary inversely with Zr_90_ (Zr in primary magmas, Zr_90_). Fraction of mantle melting in the top of the panel represents the degree of mantle melting calculated by Zr contents in primary magmas.

We use Cu/Zr ratios as a proxy to constrain the characteristics of Cu in primary arc and MORB magmas and estimate the redox states of their mantle sources. Under sulfide-absent circumstances, Zr and Cu have similar bulk partition coefficients during mantle melting and early-stage basaltic magma differentiation (see Materials and Methods and table S1). On the other hand, unlike Cu, there is no accessory Zr host in the sub-arc mantle or during early-stage mafic magma differentiation (*30*). Therefore, Cu/Zr ratios remain constant absent a sulfide phase and yet vary regularly during melting and differentiation once sulfides participate. When sulfide is present as a residual phase, Cu/Zr ratios in mantle melts increase with increasing extents of mantle melting and then remain unchanged after residual sulfide is no longer present (Fig. 1A). Furthermore, during early-stage differentiation of basaltic magma, Cu/Zr ratios would stay constant before the onset of sulfide saturation (MgO >6 wt % for arc volcanics; Fig. 1B), when Cu might then be sequestered into either a sulfide melt (*31*) or a fluid (*7*) during further magma differentiation, leading to lower Cu/Zr ratios. No matter what process is responsible for the Cu depletion in differentiated arc magma, the relatively constant Cu/Zr ratios for global arc volcanics with MgO over 6 wt % would suggest that no process significantly fractionates Cu from Zr during early-stage basaltic magma differentiation (Figs. 2B and 3B). Therefore, arc volcanics with MgO over 6 wt % could honestly record their primitive magma compositions, and thus, their Cu/Zr ratios would be a sensitive indicator of extent of melting, with more dynamic range than Cu contents alone.

Ocean ridge basalts are known to be derived by various extents of melting, reflected in major element chemistry, crustal thickness, and axial depth (*32*, *33*). Data from shallow (Reykjanes Ridge) and deep (Gakkel Ridge) ridge segments show that, during differentiation, Cu contents change little and Cu/Zr ratios decrease slightly above 8 wt % MgO. The Cu contents and Cu/Zr ratios of primitive Gakkel samples (with MgO >8 wt %) are ~70 ppm and 0.8, compared to 120 ppm and ~2 for those from the Reykjanes Ridge, consistent with other data showing greater degrees of melting in the Reykjanes Ridge (Fig. 2A and fig. S4) (*33*). In general, average Cu/Zr ratios for primitive MORB from individual ridge segments correlate inversely with axial depth and Zr_90_ [Zr contents corrected to be in equilibrium with mantle olivine of Fo_90_ (*33*), an index for extents of mantle melting; Figs. 2C and 3D], consistent with variations in temperature and extents of melting beneath global ocean ridges (*32*–*34*).

Global arc volcanics have also been shown to undergo large differences in extent of melting (*35*–*38*), and this is reflected in their Cu/Zr relationships as well. Arc volcanics with MgO over 6 wt % show constant Cu/Zr ratios due to sulfur-undersaturated early-stage magma differentiation (*7*, *31*, *39*), but Cu contents and Cu/Zr ratios vary greatly in volcanics from thin-crusted island arcs (e.g., Kermadec; Figs. 2B and 3B and fig. S4) and thick-crusted continental arcs (e.g., Cascades). This leads to a correlation of Cu/Zr in primary arc magmas with crustal thickness and Zr_90_ (Figs. 2D and 3D), consistent with an important role for the control of partial melting in Cu contents and Cu/Zr ratios in arc volcanics (Fig. 3, C and D, and fig. S5).

Three lines of evidence show that these relationships for global arcs are not a consequence of crustal processes. First, lower Cu contents for volcanics at thick-crusted arcs are documented across a large range of MgO contents (Fig. 3A). Second, the Cu/Zr versus Zr systematics of global arc segment means for arc volcanics with Mg# >60 from Turner and Langmuir (*36*), the least differentiated samples, exhibit the same relationship (fig. S6). The third line of evidence comes from comparison with MORB. Arc volcanics and MORB data have the same Cu/Zr versus Zr_90_ systematics (Fig. 3D), despite that no control by continental crust is possible at ocean ridges. Cu/Zr is thus a sensitive indicator of extents of partial melting. The global datasets of MORB and arc volcanics are derived by variable extents of melting, and the data are consistent between convergent and divergent margins.

This analysis shows that high MgO samples alone are not representative. Arc volcanics with MgO from 3 to 8 wt % (more than 65% of global arc volcanics) show systematically lower Cu contents in arcs on thick crust than those built on thinner crust, and such a trend is obscured in arc volcanics with MgO over 8 wt % (Fig. 3A). Such systematic global relationships with crustal thickness and extents of melting are consistent with a wealth of other data (*36*–*38*). Any evaluation of Cu systematics must thus take into account the consequences of different extents of melting that are critical in both ocean ridge and convergent margin settings.

These results also demonstrate, however, that Cu/Zr ratios in basalts vary similarly in ocean ridge and convergent margin settings (Fig. 3D). Since their Cu/Zr ratios are also sensitive to mantle oxygen fugacities, this would seem to imply that the oxygen fugacities during mantle melting are similar in the two settings, in accord with the conclusions of Lee *et al*. (*10*). However, Cu/Zr ratios in primitive magma are sensitive to not only oxygen fugacities but also the S contents of the mantle. Therefore, a complete evaluation requires consideration of variations in mantle sulfur contents.

### Sulfur content and speciation in the sub-arc mantle

Because of the degassing of arc magma, directly measured sulfur contents in arc volcanics underestimate the sulfur contents of parental magmas (*40*–*43*). Two alternative approaches can be used to predict primary sulfur contents. The first is the undegassed melt sulfur budget estimated from melt inclusions in primitive olivine. The second is to calculate a sulfur budget based on global sulfur emissions. Both methods lead to minimum estimates. A compilation of 66 melt inclusions in olivine with Fo higher than 85 leads to the average sulfur contents of ~2000 ppm (table S7). With a global sulfur emission mass of 1 × 10^13^ to 2 × 10^13^ g/year from subduction zone magmatism (*40*–*42*, *44*, *45*), a global arc magma production rate of 40 km^3^/km per million year (Ma) (*46*), and an arc magma density of 2500 kg/m^3^ (*47*), the second method leads to sulfur contents in the primary arc magmas ranging from 1800 to 3600 ppm (see Materials and Methods and table S8) even without considering the likely considerable amounts of S that remain locked in the arc crust.

Both methods lead to consistent estimates of sulfur contents in primary arc magmas of 2000 to 3000 ppm, comparable with previous estimates (*16*, *40*, *47*, *48*). These values exceed the sulfur contents of primary MORB magma of 800 to 1500 ppm (*40*, *41*) that can be more directly determined in undegassed glasses. Such a high sulfur content for primary arc magmas also exceeds the sulfur solubility of basaltic magma at FMQ [1200 ± 200 ppm (*23*–*25*) and ~1400 ± 300 ppm for anhydrous and hydrous magma, respectively; see Materials and Methods and table S6] and can only be achieved by higher oxygen fugacities.

The magma sulfur contents can then provide bounds on the sulfur contents of the mantle. The sub-ridge mantle contains ~200 ppm S (*49*, *50*). To produce such high sulfur contents in arc magmas from a mantle with the same sulfur content would require that all the sulfide be exhausted during melting and that extents of melting are less than 7%, which is not tenable. Arc volcanics are also known to form by extents of melting similar to or greater than that for global MORB (8 to 20%; Fig. 3D) (*33*, *35*). Using a median mantle melting degree of 15% for global arc volcanics, and still assuming that all sulfides in the mantle are extracted from the mantle to the arc magmas, the minimum sulfur contents in the sub-arc mantle can be estimated to be 300 to 450 ppm. Such estimated sulfur contents in sub-arc mantle are consistent with previous studies (*51*, *52*) and are much higher than sub-ridge mantle.

There are also clear mechanisms to increase mantle S contents in subduction settings (*15*–*17*). Both natural studies on veins in eclogite, which represent fossilized pathways for slab fluids and experimental studies, show that sulfur contents in slab-derived fluids and melts could be ~1 wt % (*53*, *54*) and ~3000 ppm (*13*, *51*, *55*, *56*). Therefore, 1 to 2.6% and ~4 to 9% contribution of slab-derived fluids and melt to the sub-arc mantle melting region would be needed to match the elevated sulfur contents, respectively, consistent with previous estimations on slab flux contributions (*38*, *57*). The slab-derived Cu enrichment, however, is minimal. With the Cu contents in fluids and slab-derived melts of ~113 ppm (sulfide-bearing vein in eclogite) (*58*) and ~30 ppm (see Materials and Methods), respectively, the sub-arc mantle Cu contents would only increase from 30 to 32 ppm with 1 to 2.6% of slab-derived fluid addition and remain unaffected with melt addition. Therefore, the slab flux likely doubles the sulfur contents of the mantle wedge while leaving Cu contents essentially unchanged (table S9).

Although slab-derived sulfur can be sulfate as documented by sub-arc mantle xenolith in Kamchatka (*13*), sulfides were found to precipitate near those sulfate-rich inclusions, with the host spinel showing increasing Fe^3+^/ΣFe ratios closer to the inclusion. Such an observation recorded the process of sulfate in the inclusions being reduced to oxidize the sub-arc mantle, which would cause a net addition of sulfide to the mineral assemblage in the sub-arc mantle during the increase of *f*o_2_ from FMQ to sulfide–sulfur oxide (*59*). Therefore, although minor amount of sulfate might be stable in the sub-arc mantle, the dominant sulfur speciation in the sub-arc mantle is still sulfide with a higher portion than the MORB mantle and would thus control the Cu systematics during melting. Upon melting, however, ferric iron would be preferentially extracted to the melt from the mantle, which would potentially oxidize the dominant sulfide extracted from the mantle to sulfate (*20*) under *f*o_2_ higher than FMQ and increase the solubility of sulfur in the melts accordingly.

### Quantitative estimation on the oxygen fugacities of primary arc magmas using Cu/Zr versus Zr

**Fig. 4.**
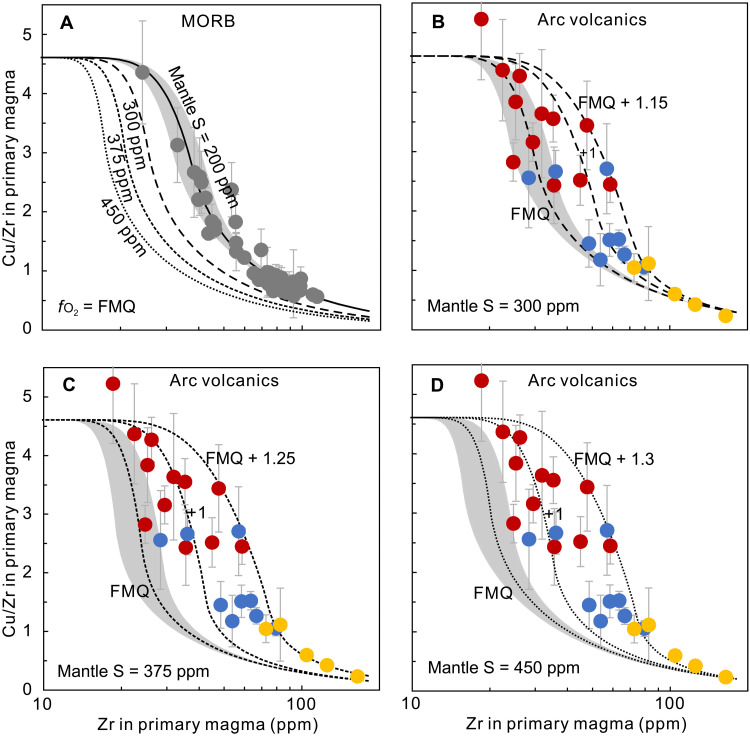
Estimated sub-arc mantle oxygen fugacities with varied mantle sulfur contents. Quantitative estimation on mantle redox states beneath (**A**) MOR and (**B** to **D**) arc with varied mantle sulfur contents using Cu/Zr versus Zr variations for primary MORB and arc magmas. (A) The black dashed lines show the mantle melting models with different mantle sulfur contents at *f*o_2_ = FMQ. Curves in (B) to (D) represent mantle melting models at different redox states (shown by numbers next to the curves) with mantle sulfur contents of 300 ppm (B), 375 ppm (C), and 450 ppm (D), respectively. Gray and colored dots represent the calculated Cu/Zr ratios in primary arc and MORB magma with 2 SE, respectively. We show the average values calculated for each point with at least three samples to be representative. The shaded areas represent the 20% variations of sulfur solubility used in the mantle melting model.

With the estimated sub-arc mantle sulfur contents, the Cu extraction efficiency can be quantitatively evaluated with melting models using Cu/Zr and Zr variations of primary arc and MORB magmas. With a mantle sulfur content of 200 ppm, global MORB data mostly plot around the modeled melting curve at FMQ (Fig. 4A). If the sub-arc mantle were also at FMQ, then the higher sulfide fraction in the sub-arc mantle would elevate the bulk *D*_Cu_ during melting, leading to lower Cu and Cu/Zr in the primary arc magma (Fig. 4A), which is not observed. To match the observed Cu/Zr and Zr variations for primary arc magma, the estimated oxygen fugacities for sub-arc mantle range from FMQ up to FMQ + 1.3 with varied mantle sulfur contents from 300 to 450 ppm (Fig. 4, B to D). Although different mantle sulfur contents would lead to varied mantle redox states beneath arcs, all models suggest that the oxygen fugacities of sub-arc mantle must be up to one log unit higher than that of sub-ridge mantle (~FMQ) (*20*–*22*).

This analysis then solves the apparent contradiction between suggested higher oxygen fugacities of the mantle at convergent margins compared to ocean ridges (*2*, *12*) and the similar Cu contents of primitive ocean ridge basalts and arc volcanics (*10*). Because of the higher sulfur contents of the sub-arc mantle, only higher oxygen fugacities during convergent margin mantle melting can lead to the overlap between MORB and arc volcanics data. Higher oxygen fugacities of the sub-arc mantle are also required simply from the higher S contents of estimated primary arc magmas alone.

The new understanding of the global systematics of Cu contents of global arc volcanics also has implications for the origin of Cu-porphyry ore deposits. Previous work has proposed that lower Cu contents in volcanics from thick-crusted arcs result from deep crustal sulfide fractionation (*10*, *19*, *39*), which would then lead to a Cu-rich source that could later be tapped for Cu-porphyries (*10*, *39*). Our study suggests instead that the low Cu contents of volcanics from thick-crusted arcs result from lower extents of melting (Fig. 3 and figs. S4 and S5), thus removing the necessity for deep crustal sulfide accumulation. More recent work has shown that even primary magmas with low Cu contents are able to generate porphyry-Cu deposits through fractionation at deep crustal levels that does not involve sulfide accumulation (*60*, *61*).

## MATERIALS AND METHODS

### Compilation of Cu dataset

The global arc data used in this study are downloaded from GEOROC (Geochemistry of Rocks of the Oceans and Continents) (http://georoc.mpch-mainz.gwdg.de/georoc/). We imported the raw data into Google Earth and selected samples located at arc fronts (fig. S2). To produce reliable dataset, we filtered the dataset following Turner and Langmuir (*36*). Data collected before 1980 and samples labeled “extensively altered” or reported loss on ignition over 2% were removed. Afterward, we selected data with Cu concentrations analyzed by inductively coupled plasma mass spectrometry (ICP-MS) or x-ray fluorescence (XRF). In addition, trace elements are included where the methods are ICP-MS, XRF, and ICP-AES (atomic emission spectrometry). The filtered “clean” dataset was regrouped and plotted on the basis of crustal thicknesses (table S1) (*36*). In particular, arc volcanics at Torishima and Tafahi volcanoes of the (S)Izu and (N)Tonga arcs are characterized by anomalously high Cu and Cu/Zr ratios among global arcs (table S4), indicating that special processes may have influenced their mantle compositions as proposed by previous studies (*62*, *63*) and lead to the inconsistent correlation with global arc dataset. Therefore, data from these two arcs are not included in the synthesis but can be found in the Supplementary Materials (fig. S7).

### Partition coefficients of Cu and Zr in silicate minerals

The *D* values of Cu used in this study are 0.05, 0.039, 0.06, and 0.19 (*28*) and those of Zr are 0.007 (*64*), 0.027 (*65*), 0.103 (*65*), and 0.06 (*66*) between olivine/melt, orthopyroxene/melt, clinopyroxene/melt, and spinel/melt, respectively. Therefore, under sulfide-absent circumstances, the bulk *D* values of Cu and Zr during mantle melting are roughly comparable (~0.05 and ~0.03, respectively; table S2), calculated with the mantle mineral assemblage by Workman and Hart (*67*). During early-stage sulfide-undersaturated magma differentiation, the *D* values of Cu and Zr in the crystallizing silicate minerals (olivine and clinopyroxene) would be roughly comparable as well. Therefore, the Cu/Zr ratio can be used as an index to trace the behavior of sulfides during mantle melting and early-stage magma differentiation.

### Mantle melting model

To quantify the sulfur behavior during mantle melting, we applied nonmodal fractional melting model for sub-arc and sub-ridge mantle (table S2) by removing equilibrium melts of each increment and changing the composition of peridotite residues (*10*). We use mineral assemblages of spinel lherzolite (57% olivine, 28% orthopyroxene, 13% clinopyroxene, and 2% spinel) from Workman and Hart (*67*) and trace element compositions of the mantle (30 ppm Cu and 6.51 ppm Zr) from Salters and Stracke (*68*) and Workman and Hart (*67*) as the initial mantle compositions. In the modeling, we use hydrous melting reactions from Gaetani and Grove (*69*) and Parman and Grove (*70*) for sub-arc mantle, and anhydrous melting reactions from Gaetani and Grove (*69*) and Falloon *et al.* (*71*) for sub-ridge mantle.

Sulfide has long been known to be partially molten and occurs as both solid [monosulfide solid solution (mss)] and liquid phases in the upper mantle at the asthenosphere temperature (*72*); however, the exact portion of each phase is not clear. The mantle temperatures responsible for the melting beneath arcs are known to be lower than that beneath ridges (*34*, *57*, *73*); thus, the portions of mss in the mantle sulfides beneath arcs should be much higher than those beneath ridges where the sulfide liquid is the dominant occurrence in the asthenosphere (*72*, *74*). Therefore, here, we assume that the ratios of sulfide liquid to mss are 1:1 and 4:1 in the mantle beneath arcs and ridges, respectively, and that their proportions in the mantle residue would be constant until total sulfide exhaustion. The partition coefficients of Cu between mss and sulfide liquid in the mantle are well constrained and range from 0.2 to 0.3 with an average of 0.25 (*27*), suggesting that the partition of Cu between the two mantle sulfide phases obeys Henry’s law. Therefore, $D_{\text{Cu}}^{\text{mss}/\text{melt}}$ can be calculated to be 250 according to$\;D_{\text{Cu}}^{\text{sulfide liquid}/\text{melt}}$ of 1000 (*26*, *27*), and the partition coefficient of Cu in bulk mantle sulfides $\left(D_{\text{Cu}}^{\text{sulfide}/\text{melt}}\right)$ during melting of sub-arc and sub-ridge mantle can be estimated to be 625 and 850, respectively.

During melting, sulfur content in each melt increment (${\left[S\right]}_{\text{melt}}^{i}$) is equal to the sulfur solubility in the melt. Therefore, the sulfur content in the mantle can be estimated by progressive removal of such fractional melt

$${\left[S\right]}_{\text{Per}}^{i+1}=\frac{M_{i}\times {\left[S\right]}_{\text{per}}^{i}-{\left[S\right]}_{\text{melt}}^{i}\times ∆M}{M_{i+1}}$$ (3)

where *M_i_* and *M*_*i*+1_ is the mass of peridotite residues at a given stage and ∆*M* is the extraction of melt between melting stage *i* + 1 and *i*. The sulfur content in the aggregated melt is equal to the sulfur solubility in the melt before sulfide exhaustion in the mantle but would be diluted afterward. The Cu and Zr contents in each melt increment can be estimated by

$$C_{L}^{i+1}=\frac{C_{\text{per}}^{i}}{\text{bulk}\;D+(1-\text{bulk}\;D)\times ∆M/M_{i}}$$ (4)

where $C_{\text{per}}^{i}$ is the concentration of Cu and Zr in the peridotite residue at stage *i*.

Therefore, the Cu concentration in the aggregated melt ${\bar{C}}_{L}^{i+1}$can be estimated by mass balance during progressive melting, which is given by

$${\bar{C}}_{L}^{i+1}=\frac{{\bar{C}}_{L}^{i}\times F^{i}+C_{L}^{i+1}\times ∆M}{F^{i+1}}$$ (5)

where *F^i^* is the weight fraction of aggregated melt at stage *i*.

### Calculation on Cu and Zr contents in primary arc and MORB magmas (Cu_90_, Zr_90_)

To compare the melt compositions (such as Cu and Zr contents) of primary magmas, we should first remove the effect of crystallization by fractionation correction. Extrapolating the melt composition in equilibrium with mantle olivine is not a fixed procedure that depends on the MgO content, where plagioclase and clinopyroxene appear in MORB and arc magmas, respectively (*33*, *35*, *36*). For anhydrous MORB magmas, liquid lines of descent provide consistent evidence that fractionation of plagioclase primarily occurs between 8 and 9 wt % MgO, mostly ~8.5 wt % MgO (*33*). For hydrous arc magmas, on the contrary, clinopyroxene occurs before plagioclase, leading to a different reference value for correction (*35*, *36*). Liquid lines of descents of arc magmas provide consistent evidence that fractionation of clinopyroxene primarily occurs below ~6 wt % MgO (*35*, *36*). We followed the approaches of Gale *et al*. (*33*) and Turner and Langmuir (*36*) by using the average of samples with MgO between 8 and 9 wt % and 5.5 and 6.5 wt % as the reference values at MgO of 8.5 and 6 wt % for MORB and arc segments, respectively. We then extrapolate these 8.5 or 6 wt % MgO equivalent compositions to primary magmas by adding equilibrium olivine (using *K*_D_^ol-liq^ [Mg − Fe] = 0.3) (*75*) to each glass composition in 1% increments until in equilibrium with olivine of Fo_90_. The corrected primary arc and MORB magma composition are shown in tables S4 and S5, respectively.

### Estimating the Cu/Zr ratios for primary arc and MORB magmas

As shown in Figs. 2B and 3B and fig. S7, Cu/Zr ratios in arc volcanics with MgO over 6 wt % remain relatively constant, consistent with sulfur-undersaturated fractionation at the early stage of arc magma differentiation (*7*, *31*, *39*), and thus, Cu/Zr ratios in primary arc magma from each arc segment can be inferred from averaging arc volcanics with MgO over 6 wt %. The Cu/Zr ratios of representative ridge segments from global ocean basins are generally constant with MgO >8 wt % (Fig. 2A and fig. S8); therefore, Cu/Zr ratios in primary magma of each ridge segment can be estimated by averaging MORB with MgO over 8 wt %, which is the method we used here.

### Estimation of sulfur solubility in the melt during mantle melting

The sulfur content at sulfide saturation (SCSS) for melt is controlled by redox state, melt composition (FeO and H_2_O contents), sulfide compositions, pressure, and temperature of the melt (*23*–*25*, *73*). Using the compositions of primitive MORB samples (Mg# from 70 to 72) from the compilation of Gale *et al*. (*29*), corrected primitive compositions of arc magma in this study, the temperature of melting region from 1300° to 1400°C for MORB (*34*) and around 1100° to 1300°C for arc (*57*, *73*), and the pressure of melting region from 1 to 2.5 GPa (*73*), the average SCSS of primitive MORB and arc melt at *f*o_2_ = FMQ are thus estimated to be 1200 ± 200 (1σ) ppm and 1400 ± 300 (1σ) ppm, respectively, assuming 4% water in primitive arc magma (*57*) (table S6). Such a calculated value is consistent with previous studies (*23*–*25*, *76*). The SCSS at higher oxygen fugacities for sub-arc mantle melting can then be calculated based on the SCSS and *f*o_2_ correlation of Jugo *et al*. (*25*) to be 2500 ± 500 ppm at FMQ + 1 and 1.25 ± 0.27 wt % at FMQ + 1.5, respectively.

### Estimation of sulfur contents in primary arc magmas

Two approaches are used to estimate the sulfur contents in primary arc magmas in this study. First, we collected the data of melt inclusions hosted in olivines from global arcs. To reduce the degassing effect on the S contents in melt inclusions, we only use the data of melt inclusions hosted in olivine with Fo over 85 (table S7). We then calculated the amount of olivine that needs to be added to melt inclusion compositions to be in equilibrium with Fo_90_ olivine and correct the sulfur contents in the melt inclusions to represent the sulfur contents in the primary arc magma accordingly (table S7). Second, the sulfur content in primary arc magma can be calculated by sulfur emission flux (*F*_s_), which has been estimated to range from 1 × 10^13^ to 2 × 10^13^ g/year (*44*, *45*). Using an arc magma volume (*V*) of 40 km^3^/km per Ma from Dimalanta *et al.* (*46*), an arc length (*L*) of 55,000 km from Stern (*77*), and a magma density (ρ) of 2500 kg/m^3^ from Scaillet *et al*. (*47*), the sulfur contents in primary arc magma ($C_{S}^{\text{melt}}$) can be estimated from 1800 to 3600 ppm (table S8) from Eq. 6

$$C_{S}^{\text{melt}}=\frac{F_{S}}{V\times L\times \rho }$$ (6)

### Estimation of Cu contribution from slab-derived melt

The Cu contents of melts from the slab can be modeled with an average MORB composition from Gale *et al*. (*29*), a mineral assemblage for the altered oceanic crust (AOC) of ~30% clinopyroxene and ~70% garnet in the slab (*78*), and the partition coefficients for Cu of 0.232 and 0.046 between clinopyroxene/melt and garnet/melt, respectively (*28*). As the behavior of Cu in the magma is mainly controlled by the sulfide portion, the role of S during slab melting has to be considered. Altered basalts are known to lose S during eruption-induced degassing and oxidative seafloor weathering processes (*79*–*81*); thus, we used an average sulfur content (870 ppm) of the AOC samples from ocean drilling holes 801C, 504B, and 1256D in the melting model (*79*–*81*). Considering such a high S content in the slab source, the slab melt would be sulfur-saturated, and thus, the sulfur contents of the melts would be the SCSS for the slab melt as well. Because of much higher S contents in the oceanic crust than mantle, the mass portion of sulfide phases in the subducted crust would be higher than that in the mantle, resulting in higher bulk *D* for Cu during oceanic crust melting (bulk *D*_Cu_ of 2.4; table S9) than that during mantle melting (bulk *D*_Cu_ of ~0.5; table S2) and thus much lower Cu contents in melts from oceanic crust than those in mantle melts (fig. S9). The maximum Cu content in slab melt that can be estimated using the maximum sulfur contents (3000 ppm) (*56*) of the slab-derived melts is ~30 ppm.

## Notes

**Correction :** In an earlier version, the authors excluded data for Ba and Ce for the El Salvador arc in Table S1 and erroneously reported the original value of Java arc as (2.82) in Table S4. Additionally, in Figure S4 panel B, the Kermadec and Cascades arc volcanics were incorrectly color-coded. Both Supplementary Tables and Figure S4 have been updated and the changes do not affect the results or conclusions of the manuscript.

## Supplementary Materials

### Other Supplementary Material for this manuscript includes the following:

Tables S1 to S10

Correction (4 May 2022):
In an earlier version, the authors excluded data for Ba and Ce for the El Salvador arc in table S1 and erroneously reported the original value of Java arc as (2.82) in table S4. Additionally, in figure S4 panel B, the Kermadec and Cascades arc volcanics were incorrectly color-coded. Both supplementary tables and figure S4 have been updated and the changes do not affect the results or conclusions of the manuscript. The original version is available here:
