# Supplementary material for: Oxidized primary arc magmas: Constraints from Cu/Zr systematics in global arc volcanics
Source: Sci Adv. 2022 Mar 23;8(12):eabk0718. doi: 10.1126/sciadv.abk0718 (PMC8942352; doi:10.1126/sciadv.abk0718)
Supplement: Supplementary file 3 — Correction (4 May 2022): In an earlier version, the authors excluded data for Ba and Ce for the El Salvador arc in table S1 and erroneously reported the original value of Java arc as (2.82) in table S4. Additionally, in figure S4 panel B, the Kermadec and Cascades arc volcanics were incorrectly color-coded. Both supplementary tables and figure S4 have been updated and the changes do not affect the results or conclusions of the manuscript. The original version is available here: [file sciadv.abk0718_sm.v1.pdf]

Science Advances
AAAS

Supplementary Materials for

Oxidized primary arc magmas: Constraints from Cu/Zr systematics in global arc volcanics

Si-Yu Zhao, Alexandra Yang Yang*, Charles H. Langmuir, Tai-Ping Zhao

*Corresponding author. Email: yangyang@gig.ac.cn

Published 23 March 2022, *Sci. Adv.* **8**, eabk0718 (2022)
DOI: 10.1126/sciadv.abk0718

The PDF file includes:

Figs. S1 to S9

Other Supplementary Material for this manuscript includes the following:

Tables S1 to S10

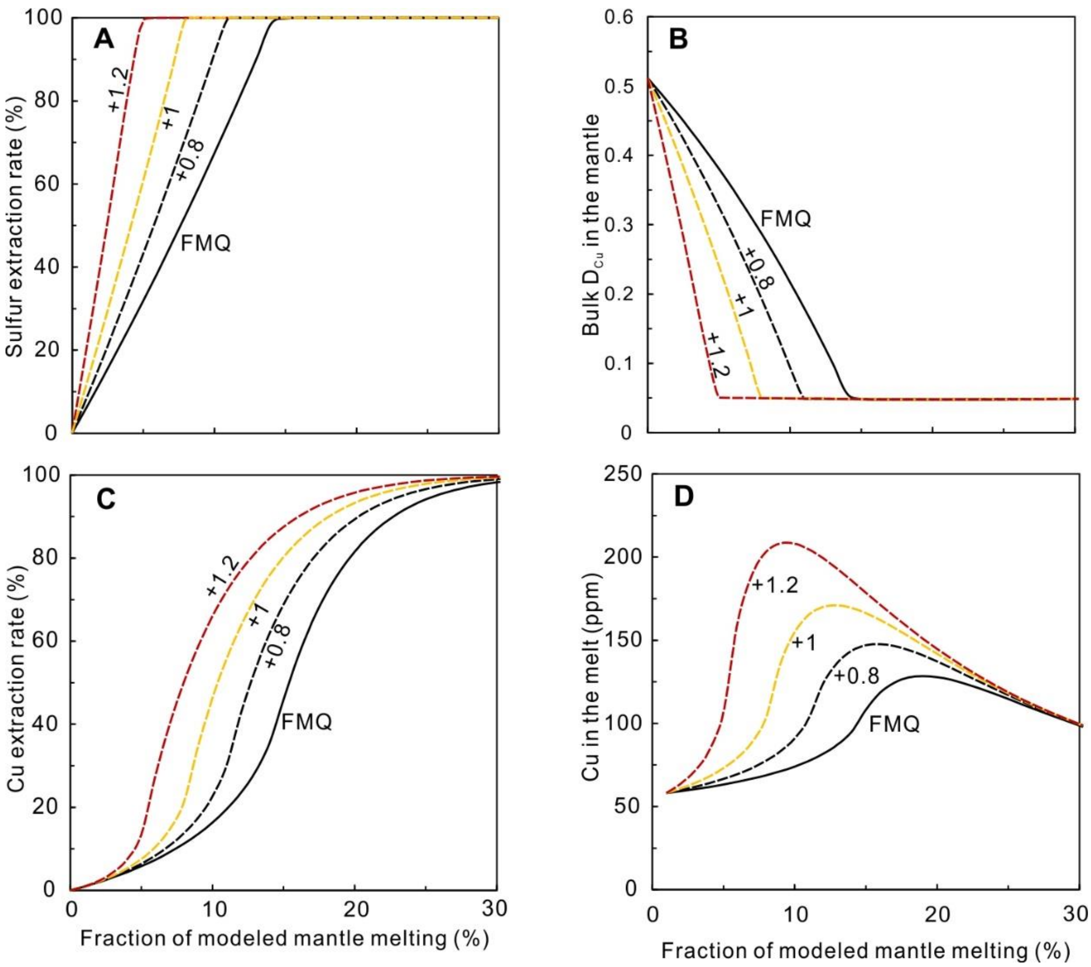

Figure S1. Behaviors of S and Cu during depleted mantle melting. Variations in the extraction rates of sulfur (**a**) and Cu (**c**) from the mantle into melts during mantle melting with different mantle oxidation states. Progressive melting depletes S in the mantle (**a**) and thus decreases bulk D_{Cu} (**b**), which accelerates the extraction rates of Cu (**c**) and enriches the melts in Cu gradually (**d**). After sulfur is exhausted, the behavior of Cu depends on silicate-melt partitioning and thus bulk D_{Cu} reach minimum values (**b**), which accelerates the extraction of Cu from residual silicate minerals. Therefore, (**d**) Cu contents in the melts increase initially and decrease shortly after sulfide exhaustion. Blank solid, and black, yellow and red dashed lines represent the modeled extraction rates for **a**) sulfur and **c**) Cu, and variations in **b**) bulk D_{Cu} and **d**) Cu contents in melts at fO_2 of FMQ, FMQ+0.8, FMQ+1 and FMQ+1.2, respectively.

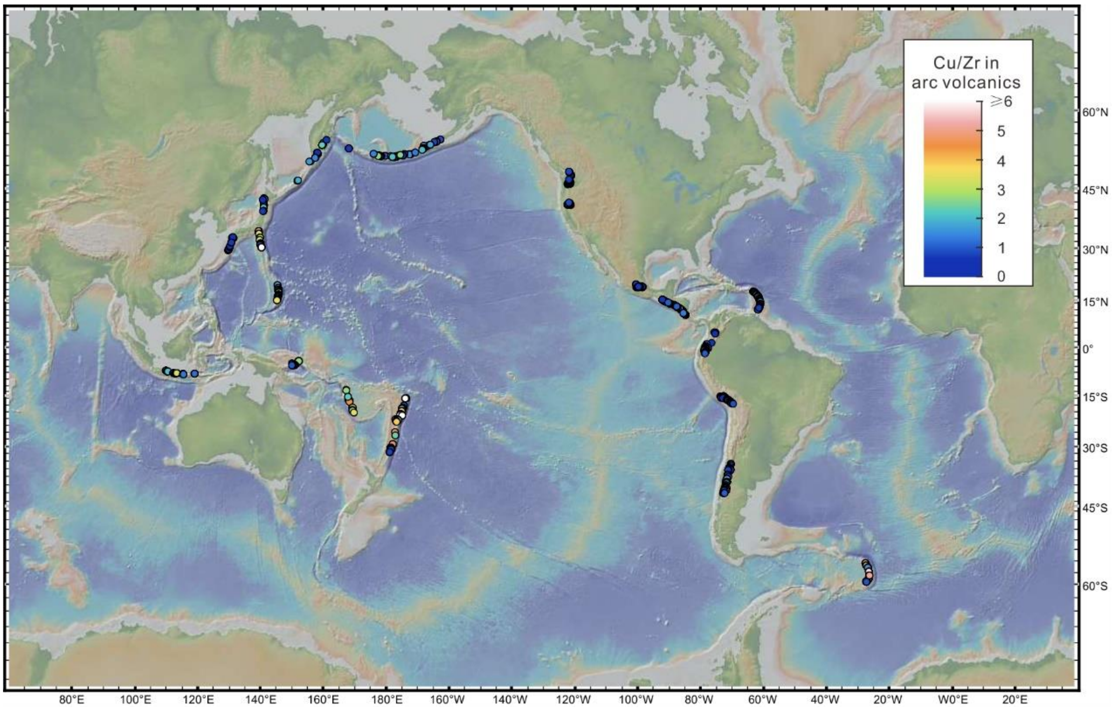

Figure S2. Map of arc frontal volcanic samples with Cu/Zr data compiled in this study.

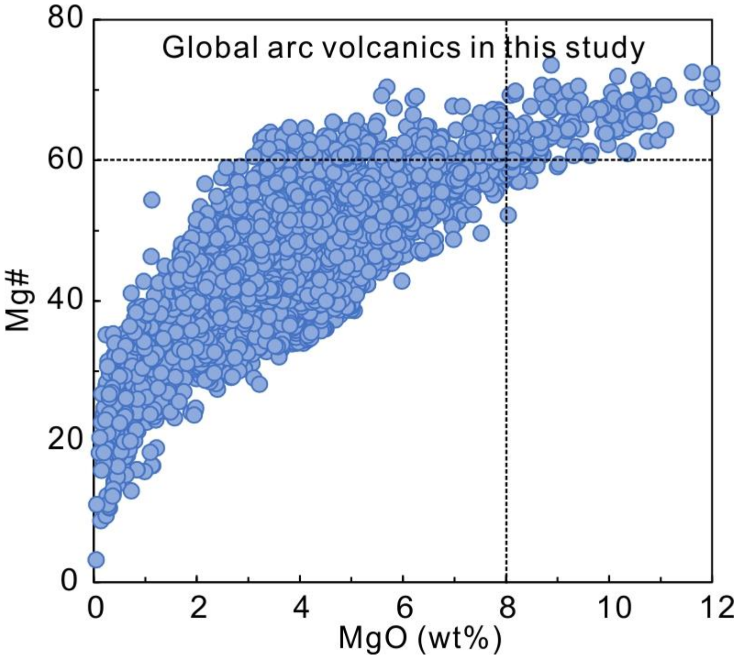

Figure S3. The Mg# vs. MgO correlations for global arc volcanics. Both Mg# >60 and MgO >8 wt.% (shown by the dashed lines) have been used to represent the primary arc magma (*33, 35-38*), and many arc samples with MgO<8 wt.% nonetheless have high Mg#.

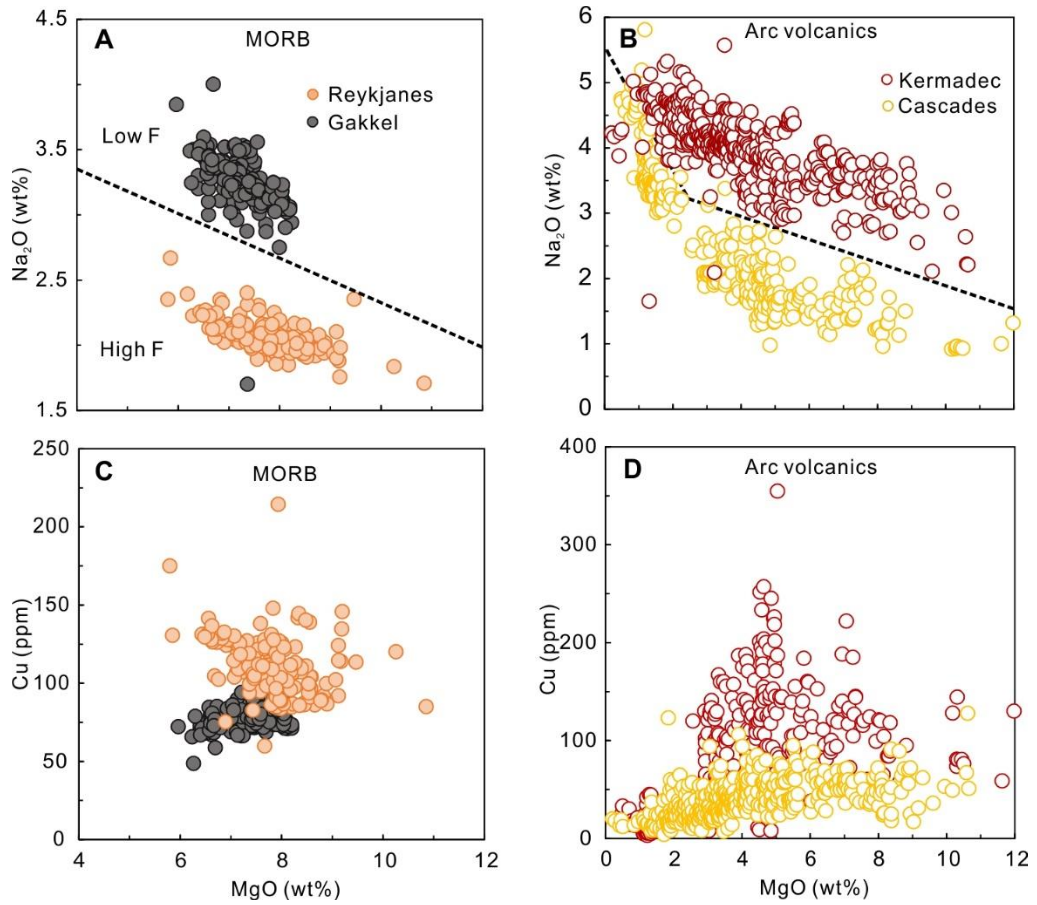

Figure S4. Na_2O and Cu vs. MgO plots for MORB and arc volcanics. Dashed lines represent partitions for variable degrees of partial melting (F). Orange and dark grey circles in **(a)** and **(c)** show the variation of Na_2O in MORB magma of the Reykjanes ridge and -6-2°E Gakkel ridge, representing the high and low melting degree endmembers, respectively. Red and yellow circles in **(b)** and **(d)** represents samples from Kermadec arc (thin-crust arc, high F) and Cascadian arc (thick-crust arc, low F), respectively. MORB data collected from Gale et al. (*29*) and Yang et al. (*83*).

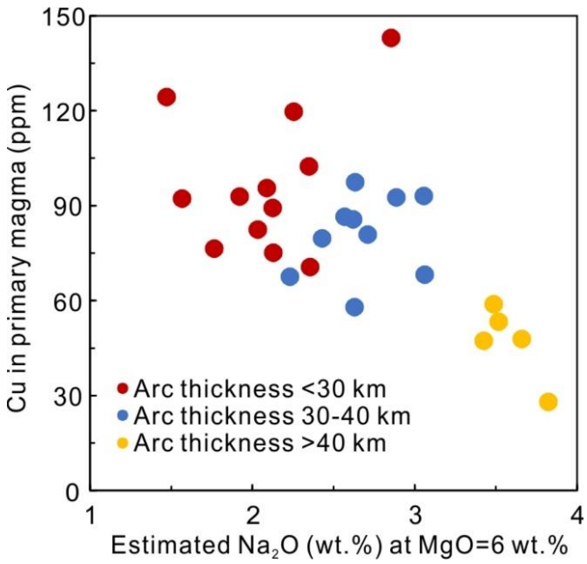

Figure S5. Cu contents in primary arc magmas vs. index for extents of mantle melting. Cu contents in primary arc magma are corrected Cu contents in magma in equilibrium with mantle olivine of Fo90 (Cu_{90}). $Na_{6.0}$ (normalized Na_2O contents for arc magmas at MgO of 6 wt.%, which is estimated as the average concentrations of Na_2O using arc volcanics with MgO between 5.5 wt.% and 6.5 wt.%, is the proxy for extents of mantle melting according to Turner and Langmuir (*36*).

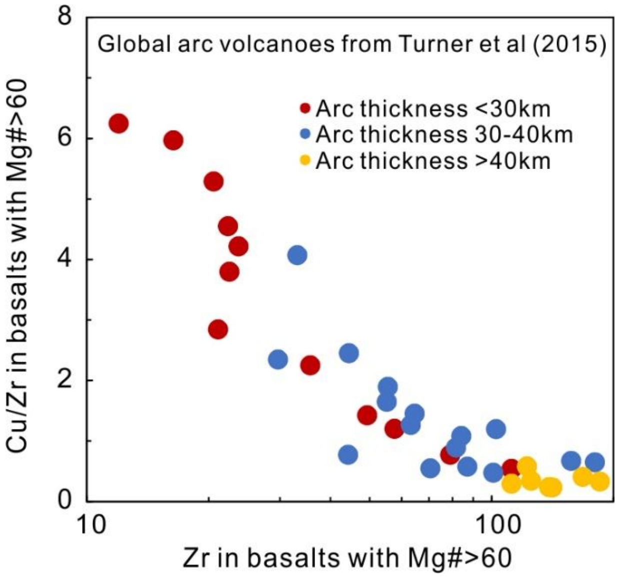

Figure S6. The variation of Cu/Zr and Zr in the least-differentiated basalts (Mg#>60) for global arc volcanoes from Turner and Langmuir (*36*). Individual dots represent the calculated Zr and Cu/Zr ratio for volcano segments from Turner and Langmuir (*36*). Red, blue and yellow circles represent volcanics from arcs with varied crustal thickness.

Figure S7. Plots of Cu/Zr vs. MgO for global arc volcanics used in this study.

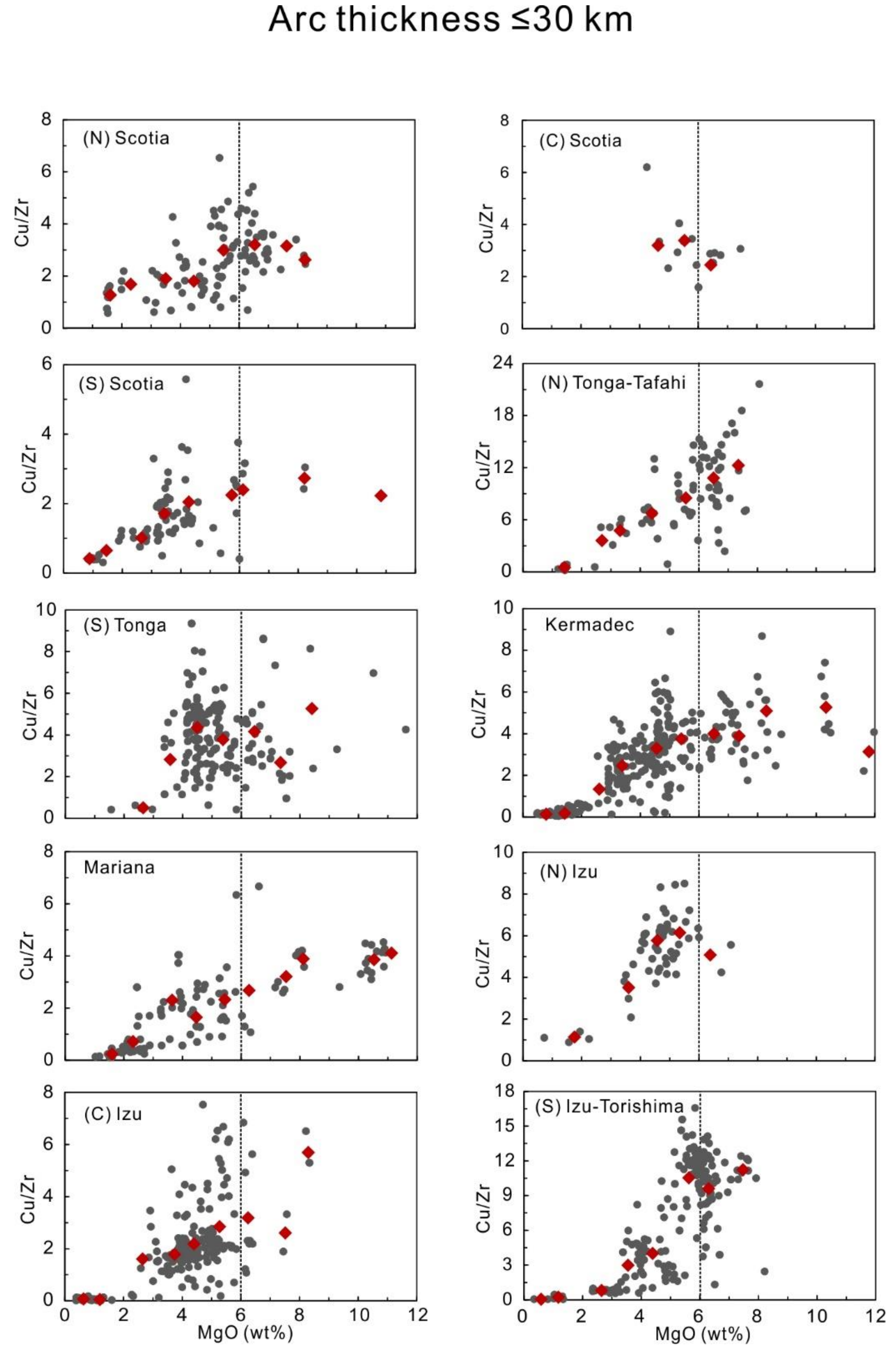

Arc thickness ≤30 km

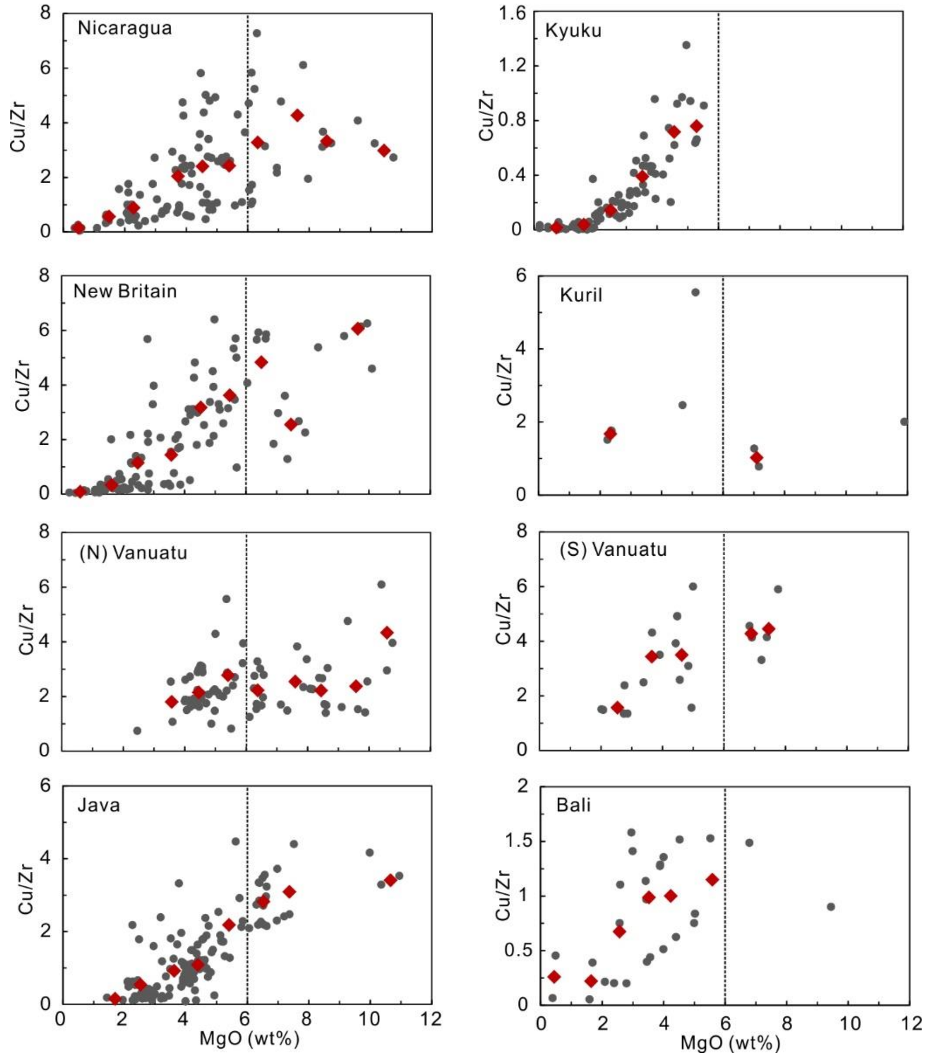

Arc thickness 30-40 km

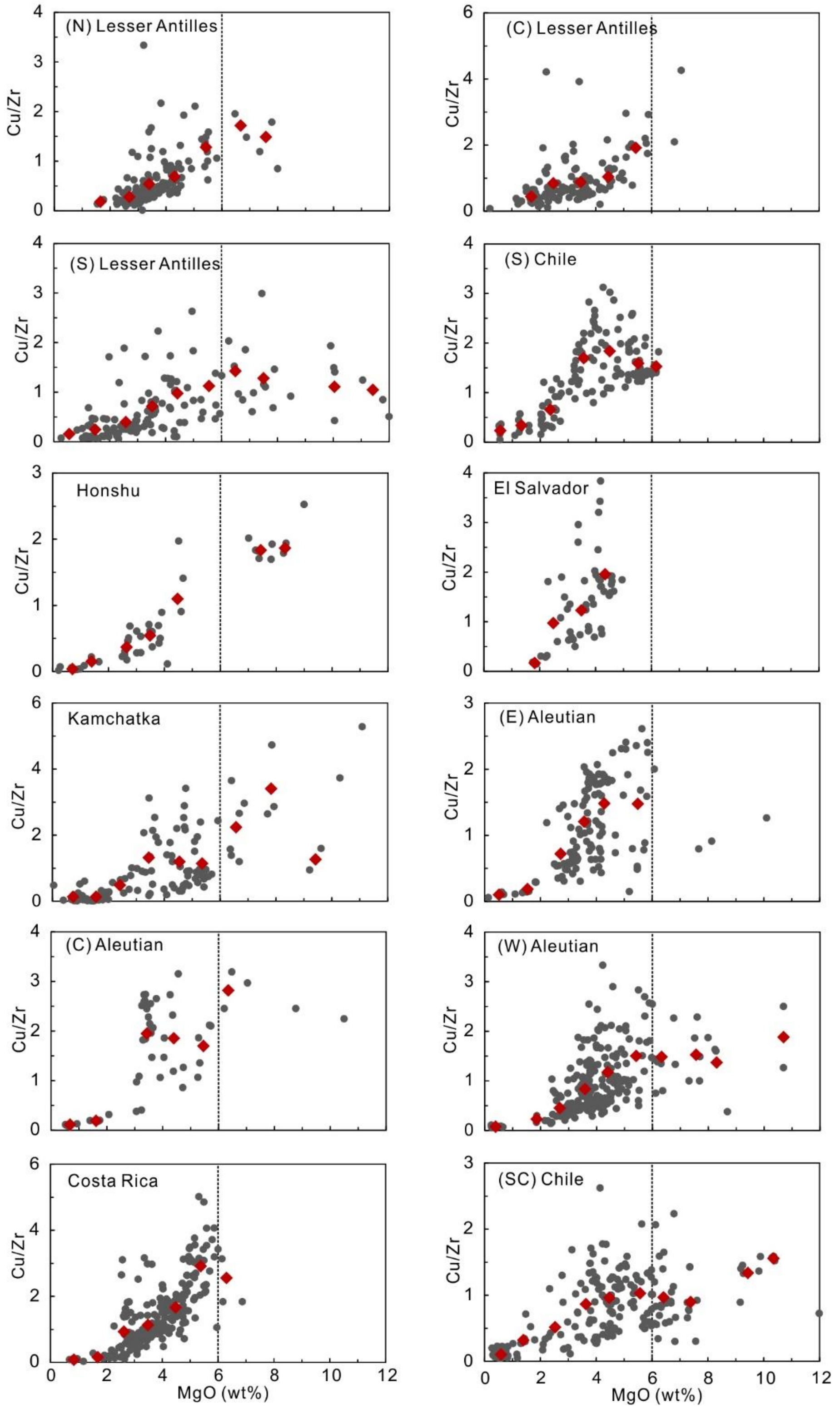

Arc thickness >40 km thick

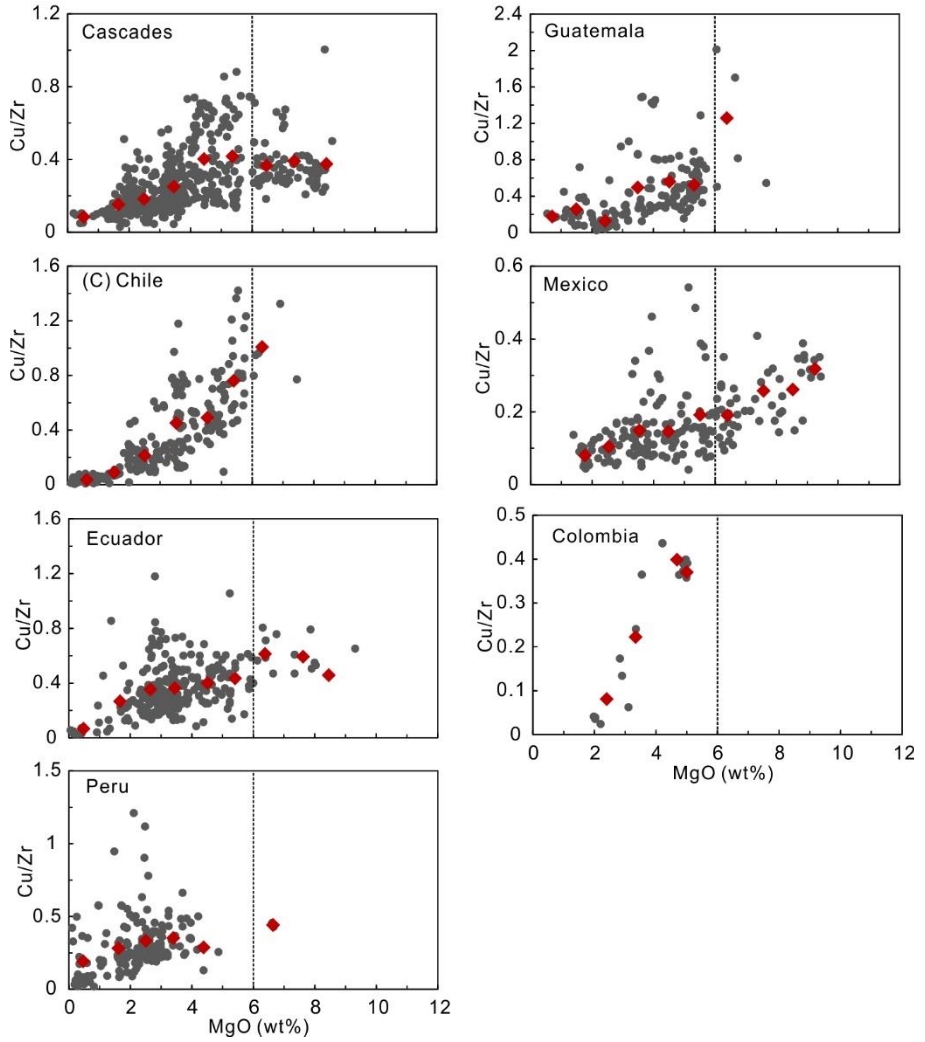

Figure S8. Examples of variations of Cu/Zr vs. MgO for MORB.

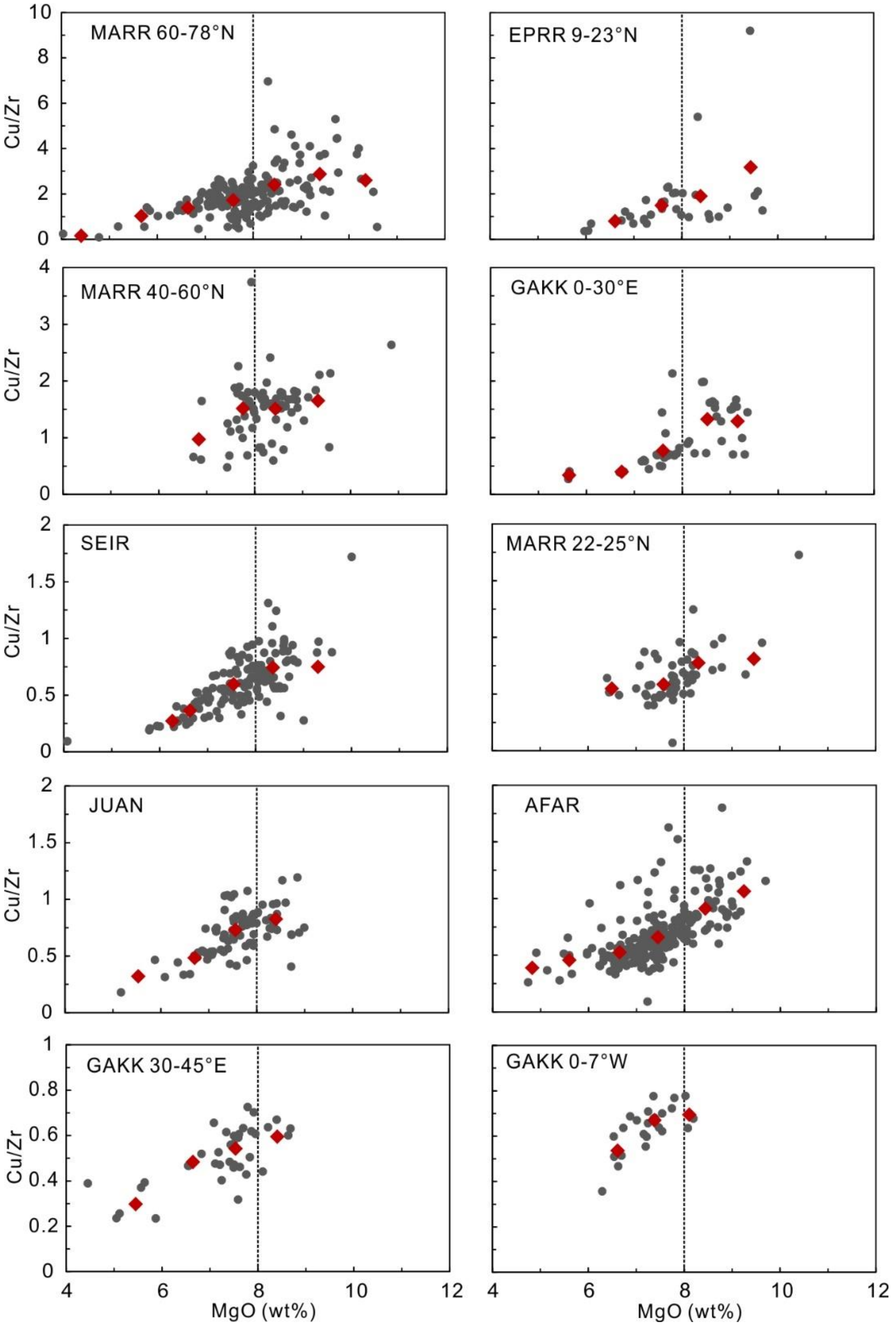

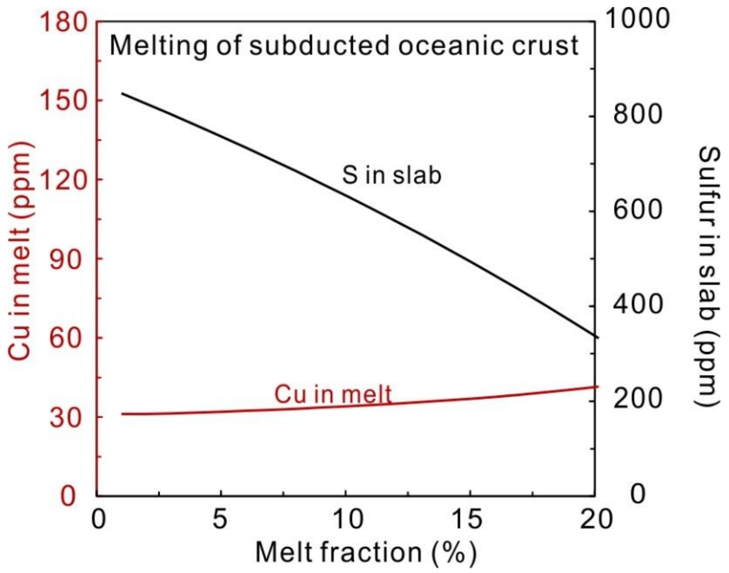

Figure S9. Quantitative constraints on the Cu variations in melt derived from melting of subducted oceanic crust. The Cu contents in the slab-derived melt (red curve) are modeled assuming that subducted oceanic crust has MORB-like compositions with 74 ppm Cu (*29*), and 870 ppm S (*79-81*). Details on the modeling can be found in Supplementary Table S9.
